# Associations of Novel and Traditional Vascular Biomarkers of Arterial Stiffness: Results of the SAPALDIA 3 Cohort Study

**DOI:** 10.1371/journal.pone.0163844

**Published:** 2016-09-29

**Authors:** Simon Endes, Seraina Caviezel, Emmanuel Schaffner, Julia Dratva, Christian Schindler, Nino Künzli, Martin Bachler, Siegfried Wassertheurer, Nicole Probst-Hensch, Arno Schmidt-Trucksäss

**Affiliations:** 1 Department of Sport, Exercise and Health, Div. Sports and Exercise Medicine, University of Basel, Switzerland; 2 Swiss Tropical and Public Health Institute, Basel, Switzerland; 3 University of Basel, Basel, Switzerland; 4 Biomedical Systems, Health & Environment Department, AIT Austrian Institute of Technology GmbH, Vienna, Austria; University of Hull, UNITED KINGDOM

## Abstract

**Background and Objectives:**

There is a lack of evidence concerning associations between novel parameters of arterial stiffness as cardiovascular risk markers and traditional structural and functional vascular biomarkers in a population-based Caucasian cohort. We examined these associations in the second follow-up of the Swiss Cohort Study on Air Pollution and Lung and Heart Diseases in Adults (SAPALDIA 3).

**Methods:**

Arterial stiffness was measured oscillometrically by pulse wave analysis to derive the cardio-ankle vascular index (CAVI), brachial-ankle (baPWV) and aortic pulse wave velocity (aPWV), and amplitude of the forward and backward wave. Carotid ultrasonography was used to measure carotid intima-media thickness (cIMT) and carotid lumen diameter (LD), and to derive a distensibility coefficient (DC). We used multivariable linear regression models adjusted for several potential confounders for 2,733 people aged 50–81 years.

**Results:**

CAVI, aPWV and the amplitude of the forward and backward wave were significant predictors of cIMT (*p* < 0.001). All parameters were significantly associated with LD (*p* < 0.001), with aPWV and the amplitude of the forward wave explaining the highest proportion of variance (2%). Only CAVI and baPWV were significant predictors of DC (*p* < 0.001), explaining more than 0.3% of the DC variance.

**Conclusion:**

We demonstrated that novel non-invasive oscillometric arterial stiffness parameters are differentially associated with specific established structural and functional local stiffness parameters. Longitudinal studies are needed to follow-up on these cross-sectional findings and to evaluate their relevance for clinical phenotypes.

## Introduction

Cardiovascular diseases (CVDs) are among the main causes of death globally, accounting for one third of all deaths [1]. Arterial stiffness is one of the main independent predictors of CVD and CVD events [2,3] and is an early subclinical sign of grave cardiovascular alterations [4]. Novel non-invasive measurements of arterial stiffness are now available to detect early vascular aging in asymptomatic people [4].

Pulse wave velocity (PWV) measurements, such as the cardio-ankle vascular index (CAVI) or brachial-ankle PWV (baPWV), are commonly used as surrogate measures of arterial stiffness [2]. Novel pulse wave analysis (PWA) methods allow detailed analyses of pulse wave characteristics in terms of aortic pulse wave velocity (aPWV) and amplitude of the forward and backward travelling wave, both of which are associated with CVD risk and modifications of the vasculature [5]. Furthermore, it is now feasible to derive aPWV from oscillometric blood pressure measurements using validated transfer functions, such as the ARCSolver algorithm [6]. aPWV is regarded as the most clinically relevant PWV measurement, due to its close physiological relationship to left ventricular mass and cardiac afterload and due to the pathophysiological consequences of increased arterial stiffness on the heart [2].

Novel parameters of arterial stiffness based on PWV measurement, such as CAVI or baPWV, and PWA parameters, such as estimated aPWV and wave reflection characteristics (amplitude of the forward and backward wave), have not yet been studied in a Caucasian population-based cohort. In particular, there is lack of evidence regarding their association with traditional structural (carotid intima-media thickness (cIMT), lumen diameter (LD)) and functional (distensibility coefficient (DC)) vascular biomarkers. Therefore, we examined whether these novel biomarkers of arterial stiffness are associated with traditional and well-established structural and functional markers of local stiffness in a cohort of older adults.

## Materials and Methods

### Study design and participants

Data were derived from the Swiss Cohort Study on Air Pollution and Lung and Heart Diseases in Adults (SAPALDIA), an ongoing multi-center cohort study initiated in 1991 among randomly selected adults (18 to 65 years, N = 9,651) [7]. The present analyses included all those who participated in the arterial stiffness and carotid ultrasound measurements in the second follow-up assessment (SAPALDIA 3, N = 2,733, study flow chart, supplemental S1 Fig). The study complies with the Declaration of Helsinki. Ethical clearance was obtained from the respective cantonal ethical committees (Aargau, Basel, Geneva, Grisons, Ticino, Valais, Vaud and Zurich) and participants gave written informed consent, according to their preferences; either globally for all examinations or separately for single assessments. Ethical approval and participant consent do not allow for public sharing of data (http://www.sapaldia.ch).

### Pulse wave velocity measurement

In SAPALDIA 3, arterial stiffness was measured non-invasively by baPWV and CAVI using a VaSera VS-1500N vascular screening system (Fukuda Denshi, Tokyo, Japan). The degree of reproducibility between two measurements within a 90-day period is high in the study population, with a low mean coefficient of variation of 3.9% for baPWV and 4.4% for CAVI; high intraclass-correlation coefficients of 0.9 and 0.8, respectively; and low mean differences in percent of the respective mean for baPWV (0.9%) and CAVI (1.7%) [8]. The baPWV and CAVI measurement is based on a common blood pressure cuff method, with occlusive cuffs on each upper arm and above each ankle, electrocardiogram electrodes at each wrist, and a phonocardiogram on the sternal border in the second intercostal space [8]. All measurements were performed following a standardized protocol, under non-fasting conditions in a quiet room with constant temperature and with participants in supine position after 10 minutes of rest. Participants had been asked to refrain from caffeine, alcohol, smoking, and exercise for at least 12 hours prior to examination. baPWV was calculated automatically by the VSS-10 software (Fukuda Denshi, Tokyo, Japan) by dividing the arterial length between the sites of interest by the time delay of the pulse wave. The arterial length was calculated by a height-based formula and the time delay of the pulse wave was determined by a foot-to-foot-method. CAVI was then mathematically derived by incorporating the blood pressure independent β-stiffness index [9], making CAVI less dependent on the blood pressure at the time of measurement.

### Pulse wave analysis

On the basis of these peripheral recordings, PWA was performed retrospectively using the ARCSolver algorithm (AIT Austrian Institute of Technology GmbH, Vienna, Austria) in order to derive aPWV for 1,267 participants as a surrogate marker of aortic stiffness, as well as central systolic and diastolic blood pressure and the amplitude of the forward and backward wave. PWA analysis was performed on a subsample of the study population (N = 1,267) that showed no statistically significant differences from the rest of the study sample (N = 2,733) in the main covariates of interest (age, sex, body mass index, mean arterial pressure, heart rate, and pack-years of smoking). The ARCSolver method is a mathematical procedure for calculating aortic stiffness and central hemodynamic measures from peripheral pulse waves and blood pressure measurements at the brachial artery using a common occlusive cuff [10]. We have recently proven the feasibility of this method in combination with the VaSera VS-1500N device [11].

### Carotid ultrasound

Following a standardized protocol, bilateral ultrasound B-mode scans of the common carotid artery were taken by trained sonographers using the UF-870 ultrasound system (Fukuda Denshi, Tokyo, Japan). Previous analyses of this study population have proven excellent reproducibility of structural parameters, with a mean coefficient of variation of 3.98% and an intraclass correlation coefficient of 0.89 for cIMT and 1.6% and 0.97 for LD, respectively, as well as good reproducibility of functional indices (DC: coefficient of variation 12.14%, intraclass correlation coefficient 0.77) [12]. Detailed information on the analysis system and examination procedure can be retrieved elsewhere [12–14]. In brief, ultrasound scans were analyzed by certified readers using the B-mode image analysis program, DYnamic ARtery Analysis (DYARA). Readers evaluated carotid structure in a standardized 1 cm segment across at least one heart cycle [13]. Within the DYARA interface, B-mode image sequences are contrasted with the virtual M-mode. The software automatically assesses the lumen-intima to media-adventitia layer of the far wall (cIMT) and the media-adventitia interface of the far wall to the media-adventitia interface of the near wall (outer LD) in the common carotid artery along the 1 cm arterial wall segment proximal to the carotid bifurcation in every time frame. DYARA offers the possibility to manually correct the measurement in case the automatic detection fails and reliably analyzes B-mode sequences with varying quality over several heart cycles [13]. Systolic (SBP) and diastolic blood pressure (DBP) were measured at the upper arm of the respective side with an OMRON 705IT device (OMRON Healthcare, Kyoto, Japan) immediately after measuring cIMT. DC is a measure of local arterial stiffness and was calculated as ((2 × deltaLD × dLD)+(deltaLD)^2^)/(PP× dLD^2^), where deltaLD is the systolic-diastolic lumen diameter difference, dLD the diastolic lumen diameter, and PP the pulse pressure [15].

### Statistical analyses

Unless stated otherwise, data are expressed as mean (standard deviation (SD)). We used Pearson’s correlation coefficients for bivariate analyses. In multivariable linear regression models, the associations of either cIMT, LD or DC as outcomes with either CAVI, baPWV, aPWV or amplitude of the forward or backward wave as main predictors were adjusted for age, sex, body mass index, heart rate, mean arterial pressure ((2 x DBP)+SBP)/3, doctor diagnosed cardiovascular disease assessed by questionnaire (angina, arrhythmia, heart failure, myocardial infarction, stroke), doctor diagnosed diabetes mellitus, and pack-years of smoking. All statistical analyses were performed using the statistical software STATA (StataCorp LP, College Station, USA) and *p* <0.05 was defined as statistical significance.

## Results

Table 1 presents the study population’s main characteristics, stratified by sex. The study population (N = 2,733) was not significantly different from the rest of the SAPALDIA 3 cohort (N = 6,088) in terms of the main predictors of age, sex, body mass index, mean arterial pressure, heart rate, and pack-years of smoking. The threshold for the highest tertile of CAVI was 9 [no unit] (N = 919), for baPWV 14.5 m/s (N = 903) and for cIMT 0.8 mm (N = 877), respectively, reflecting increased cardiovascular risk [16–19]. In total, 365 (13%) reported doctor-diagnosed CVD, defined as having been diagnosed for either angina, arrhythmia, heart failure, myocardial infarction, or stroke. One-hundred and eighty-five (7%) had doctor-diagnosed diabetes mellitus. CAVI (8.59 vs 8.96), baPWV (13.77 vs 14.36 m/s) and cIMT (0.73 vs 0.77 mm) were significantly higher for people with doctor-diagnosed CVD (all *p* < 0.001).

**Table 1:** Study population’s main characteristics by sex.

Characteristics	Units	N	Male	Female	*p*
**Age**	years	2,733	63 (8.0)	63 (8.0)	0.38
**Sex**	N (%)	2,733	1334 (49)	1399 (51)	
**Heart rate**	bpm	2,733	62 (10)	62 (10)	0.32
**BMI**	kg/m^2^	2,733	26.9 (3.5)	25.3 (4.5)	<0.001
**baPWV**	m/s	2,733	14.0 (2.5)	13.7 (2.5)	<0.05
**CAVI**	no unit	2,733	8.8 (1.2)	8.5 (1.1)	<0.001
**MAP**	mmHg	2,733	103 (13)	101 (14)	0.02
**aPWV**	m/s	1,267^a^	9.5 (1.5)	9.4 (1.6)	0.41
**Amplitude forward wave**	mmHg	1,267 ^a^	24.3 (5.8)	25.3 (6.8)	<0.05
**Amplitude backward wave**	mmHg	1,267 ^a^	15.1 (4.7)	16.5 (5.2)	<0.001
**cIMT**	mm	2,733	0.76 (0.13)	0.72 (0.12)	<0.001
**DC**	1/kPa	2,733	0.015 (0.005)	0.016 (0.006)	<0.001
**LD**	mm	2,733	7.95 (0.85)	7.20 (0.73)	<0.001
**Smoking**	pack-years	2,733	16.4 (23.1)	9.5 (16.7)	<0.001
**Doctor-diagnosed CVD**	Yes (N (%))	2,733	Yes 223 (17)	Yes 142 (10)	Overall <0.001
**Doctor-diagnosed diabetes**	Yes (N (%))	2,733	Yes 115 (9)	Yes 69 (5)	Overall <0.001

aPWV, aortic pulse wave velocity; baPWV, brachial-ankle pulse wave velocity; BMI, body-mass index; CAVI, cardio-ankle vascular index; cIMT, carotid intima-media thickness; CVD, cardiovascular disease; Doctor-diagnosed CVD: angina, arrhythmia, heart failure, myocardial infarction, or stroke assessed by questionnaire; DC, distensibility coefficient; LD, lumen diameter; MAP, mean arterial pressure. Values are mean (standard deviation) or N (%). *p*-value denotes sex comparisons using t-tests.

^a^Digital data to perform wave analysis were available only from a subsample.

### Bivariate associations

**Fig 1:**
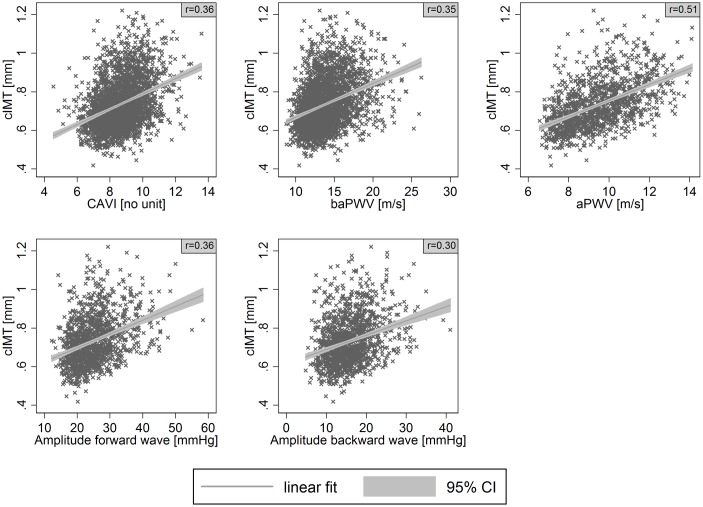
Association of carotid intima-media thickness (cIMT) with cardio-ankle vascular index (CAVI), brachial-ankle pulse wave velocity (baPWV), aortic pulse wave velocity (aPWV) or amplitude of the forward and backward wave. Scatter plots with linear prediction line (linear fit) and 95% confidence interval (95% CI). r denotes Pearson’s correlation coefficient.

**Fig 2:**
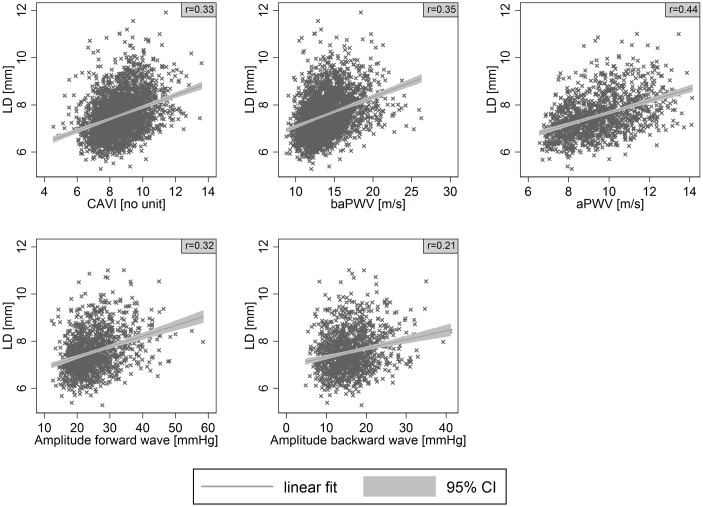
Association of lumen diameter (LD) with cardio-ankle vascular index (CAVI), brachial-ankle pulse wave velocity (baPWV), aortic pulse wave velocity (aPWV) or amplitude of the forward and backward wave. Scatter plots with linear prediction line (linear fit) and 95% confidence interval (95% CI). r denotes Pearson’s correlation coefficient.

**Fig 3:**
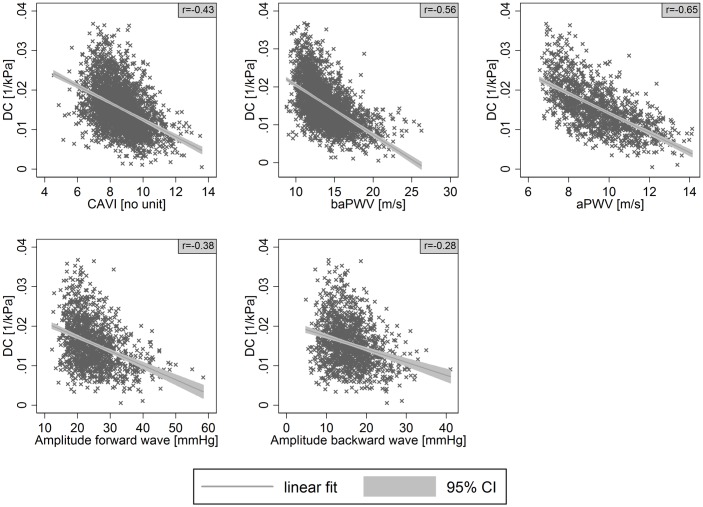
Association of distensibility (DC) with cardio-ankle vascular index (CAVI), brachial-ankle pulse wave velocity (baPWV), aortic pulse wave velocity (aPWV) or amplitude of the forward and backward wave. Scatter plots with linear prediction line (linear fit) and 95% confidence interval (95% CI). r denotes Pearson’s correlation coefficient.

CAVI was moderately and positively associated with cIMT (r = 0.36) and LD (r = 0.33), and negatively associated with DC (r = -0.43) in bivariate analyses (Figs 1–3). baPWV showed moderate positive associations with cIMT (r = 0.35) and LD (r = 0.35) and a strong negative association with DC (r = -0.56). aPWV showed a strong bivariate association with cIMT (r = 0.51), LD (r = 0.44), and DC (r = -0.64). The bivariate associations of cIMT, LD, and DC with the amplitude of the forward wave were moderate (r = 0.36, 0.32, -0.38); associations with the backward wave were weak (r = 0.30, 0.21, -0.28), with the same directions as for the other parameters of interest.

### Multivariable regression analyses

Multivariable regression analyses, adjusted for several potential confounders, revealed that CAVI, aPWV, and the amplitude of the forward and backward wave were significant predictors of cIMT (all *p* < 0.001) (Table 2). aPWV and the amplitude of the forward wave accounted for the highest part of the cIMT variance (PWA covariate R-squared 3%) compared to the other PWA predictors. All PWA parameters were significantly associated with LD (all *p* ≤ 0.001), with aPWV and the amplitude of the forward wave accounting for the highest proportion of variance compared to the other PWA predictors (PWA covariate R-squared 2%). Only CAVI and baPWV were significant predictors of DC in the multivariable regression models (all *p* < 0.001), accounting for 0.3% of the DC variance. aPWV was the strongest predictor of cIMT according to standardized beta coefficients (0.94), followed by age (0.35), and also the strongest predictor of LD (0.66), followed by sex (0.41) (data for age and sex not shown). Age was the strongest predictor of cIMT in the other models (standardized beta coefficients 0.37–0.42). Sex was the strongest predictor of LD in the other models (standardized beta coefficients 0.31–0.42). Age was the strongest predictor of DC in all models (standardized beta coefficients 0.42–0.50). The results of sensitivity analyses excluding participants with CVD were not materially different.

**Table 2:** Adjusted estimates of the effects of cardio-ankle vascular index (CAVI), brachial-ankle pulse wave velocity (baPWV), aortic pulse wave velocity (aPWV), and amplitude of the forward and backward wave on carotid intima-media thickness (cIMT), lumen diameter, and distensibility.

Stiffness parameter	Coefficient	*p*	Beta	N	Model R-squared	PWA covariate R-squared
**cIMT [mm]**						
CAVI [no unit]	0.0109	<0.001	0.10	2733	0.26	0.005
baPWV [m/s]	0.0022	0.092	0.04	2733	0.26	0.001
aPWV [m/s]	0.0765	<0.001	0.94	1267	0.31	0.033
Amplitude forward wave [mmHg]	0.0048	<0.001	0.24	1267	0.31	0.034
Amplitude backward wave [mmHg]	0.0037	<0.001	0.15	1267	0.29	0.015
**Lumen diameter [mm]**						
CAVI [no unit]	0.0826	<0.001	0.11	2733	0.38	0.007
baPWV [m/s]	0.0305	<0.001	0.09	2733	0.38	0.003
aPWV [m/s]	0.3781	<0.001	0.66	1267	0.42	0.016
Amplitude forward wave [mmHg]	0.0251	<0.001	0.18	1267	0.43	0.019
Amplitude backward wave [mmHg]	0.0150	0.001	0.09	1267	0.41	0.005
**Distensibility [1/kPa]**						
CAVI [no unit]	-0.0004	<0.001	-0.07	2733	0.49	0.003
baPWV [m/s]	-0.0002	<0.001	-0.10	2733	0.49	0.003
aPWV [m/s]	-0.0003	0.470	-0.07	1267	0.50	0.000
Amplitude forward wave [mmHg]	0.0000	0.461	-0.02	1267	0.50	0.000
Amplitude backward wave [mmHg]	0.0000	0.174	0.03	1267	0.50	0.001

Adjustment by age, sex, body mass index, heart rate, mean arterial pressure, doctor-diagnosed cardiovascular disease (angina, arrhythmia, heart insufficiency, myocardial infarction, stroke), doctor-diagnosed diabetes mellitus, pack-years of smoking. PWA covariate R-squared denotes the proportion of variance explained by the respective predictor alone. Beta denotes standardized regression coefficients.

## Discussion

Cross-sectional results from a Swiss population of older adults show that novel non-invasive oscillometric arterial stiffness and wave reflection parameters are significantly associated with established structural and functional local stiffness parameters. aPWV had the strongest association with cIMT and LD. Only CAVI and baPWV were significantly associated with DC in multivariable regression models adjusted for several potential confounders. To the best of our knowledge, this is the first epidemiological study comparing the novel arterial stiffness parameters, CAVI, baPWV, estimated aPWV, and amplitude of the forward and backward wave, based on their associations with established local stiffness and structural parameters used for cardiovascular risk assessment and prediction.

A one unit increase in CAVI was associated with a cIMT increase of 0.01 mm; a 1 m/s increase in baPWV and aPWV was associated with a cIMT increase of 0.002 mm and 0.08 mm, respectively; and a 1 mmHg increase of the forward and backward wave was associated with a cIMT increase of 0.005 and 0.004 mm, respectively. Given that the risk for future cardiovascular events increases by 16% per 0.1 mm increment in cIMT, according to a large meta-analysis of 16 cohorts [20], every increase in cIMT associated with an increase in arterial stiffness needs to be considered in view of future cardiovascular health. To date, there are no prospective cohort studies concerning associations of LD and DC with future cardiovascular events that could help put our results in perspective.

The level of evidence concerning the usefulness of carotid ultrasonography-based measurements of cIMT for primary and secondary CVD prevention has recently been classified with the highest grade, A [21]. However, it has been shown that the prognostic value of cIMT measurement in the common carotid artery concerning myocardial infarction or stroke [22] and in patients with diabetes mellitus [23] does not add incremental value to the Framingham risk score. cIMT features a valuable non-invasive surrogate measure of the subclinical progression of atherosclerosis and CVD risk, however, with limited reproducibility in daily practice if standardized protocols are missing [24].

Oscillometrically measured arterial stiffness and wave reflection parameters are regarded as easily applicable tissue biomarkers and as cumulative measures of several cardiovascular risk factors and their influence on the vasculature with ageing [4]. Both CAVI and baPWV represent mixed measures of central and peripheral arterial stiffness as the underlying PWV relates to elastic and muscular type arteries measured over several arterial segments [21]. CAVI is less dependent on blood pressure at the time of measurement compared to common PWV measurements, due to the mathematical incorporation of the blood pressure-independent β-stiffness index [25]. Both CAVI and baPWV have been shown to be valid biomarkers of CVD risk, outcomes, and lifestyle modification [26,27,17,28–30] with good correlations to aortic stiffness [31,32]. The correlation between both CAVI and baPWV with aPWV was strong (r = 0.64 and r = 0.72) in our data (S2 Fig). In our study, only CAVI (but not baPWV) was significantly associated with cIMT after full adjustment, however, both were significantly associated with LD and DC. This adds to the evidence that CAVI may be superior to baPWV as a predictor of atherosclerosis progression [33–35].

aPWV estimated from peripheral blood pressure measurement, using a transfer function like the ARCSolver algorithm, has shown good agreement with invasively measured aPWV—the gold-standard for aortic stiffness measurement [36]. Furthermore, estimated aPWV was a better predictor of cardiac load and hypertensive target organ damage than carotid-femoral PWV, similar to invasively measured aPWV [36]. Indices of wave reflections derived by wave separation analyses, like the amplitude of the forward and backward wave, are important determinants of central augmentation affecting left ventricular function and, thus, cardiac health [37].

Standardized beta regression coefficients give the relative strength of the various predictors within a model. Our analyses showed that aPWV accounts for a certain amount of variance of cIMT and LD beyond the common underlying age factor, according to the beta coefficients of the multivariable regression analyses. Age is usually the strongest predictor of structural and functional markers of arterial stiffness [4], which was true for CAVI, baPWV, and amplitude of the forward and backward wave, and for all main predictors in the DC models. This points to the potential of aPWV estimation using a transfer algorithm as an indicator of carotid and cardiac health.

### Strength and limitations

This is the first population-based Caucasian cohort comparing the novel non-invasive arterial stiffness indices CAVI and baPWV with traditional markers of structural and functional local stiffness. So far, CAVI and baPWV have mainly been used among Asian populations, reducing the possibility of extrapolating the evidence to other ethnicities [21]. Furthermore, estimated aPWV using a transfer algorithm and the wave reflection parameters amplitude of the forward and backward wave and their associations with cIMT, LD and DC have not been analyzed before in a sample representative of the general population. The cross-sectional study design does not allow us to answer questions about causality or about the parallel progression of these novel PWA indices with local stiffness parameters and the predictability of cardiovascular events. Although the aPWV and wave reflection data were restricted to a subsample of the study population, it was large enough to draw population-based conclusions and did not show a statistically significant difference in terms of the main participants’ characteristics.

## Conclusions

We demonstrated that novel non-invasive oscillometric arterial stiffness parameters are associated with established structural and functional local stiffness parameters. These parameters should not be used interchangeably, as these cross-sectional data point to a different predictive value of each of the parameters analyzed. Longitudinal studies are needed to follow-up on these cross-sectional findings and to evaluate their relevance for clinical phenotypes. Since arterial stiffness integrates the biological burden of various risk factors on the vasculature over a long period of time and is a non-invasive, easily applicable measure, it might have the potential to improve cardiovascular risk assessment in daily clinical practice.

## Supporting Information

S1 Fig Study flow chart. (TIF)

S2 Fig Association of cardio-ankle vascular index (CAVI) and brachial-ankle pulse wave velocity (baPWV) with aortic pulse wave velocity (aPWV). Scatter plots with linear prediction line (linear fit) and 95% confidence interval (95% CI). r denotes Pearson’s correlation coefficient.
(TIF)

S1 File Full SAPALDIA acknowledgement. (DOC)

## References

[1] World Health Organization (WHO), editor. The World Health Report 2003—Shaping the Future [Internet]. 2003. Available: http://www.who.int/whr/2003/en/index.html

[2] Laurent S, Cockcroft J, Van Bortel L, Boutouyrie P, Giannattasio C, Hayoz D, et al Expert consensus document on arterial stiffness: methodological issues and clinical applications. Eur Heart J. 2006;27: 2588–2605. 10.1093/eurheartj/ehl254 17000623

[3] Mattace-Raso FUS, van der Cammen TJM, Hofman A, van Popele NM, Bos ML, Schalekamp MADH, et al Arterial stiffness and risk of coronary heart disease and stroke: the Rotterdam Study. Circulation. 2006;113: 657–663. 10.1161/CIRCULATIONAHA.105.555235 16461838

[4] Nilsson PM, Boutouyrie P, Laurent S. Vascular Aging A Tale of EVA and ADAM in Cardiovascular Risk Assessment and Prevention. Hypertension. 2009;54: 3–10. 10.1161/HYPERTENSIONAHA.109.129114 19487587

[5] Parragh S, Hametner B, Bachler M, Weber T, Eber B, Wassertheurer S. Non-invasive wave reflection quantification in patients with reduced ejection fraction. Physiol Meas. 2015;36: 179–190. 10.1088/0967-3334/36/2/179 25571922

[6] Wassertheurer S, Kropf J, Weber T, van der Giet M, Baulmann J, Ammer M, et al A new oscillometric method for pulse wave analysis: comparison with a common tonometric method. J Hum Hypertens. 2010;24: 498–504. 10.1038/jhh.2010.27 20237499 PMC2907506

[7] Martin BW, Ackermann-Liebrich U, Leuenberger P, Künzli N, Stutz EZ, Keller R, et al SAPALDIA: methods and participation in the cross-sectional part of the Swiss Study on Air Pollution and Lung Diseases in Adults. Soz-Präventivmedizin. 1997;42: 67–84. 10.1007/BF01318136 9151378

[8] Endes S, Caviezel S, Dratva J, Schaffner E, Schindler C, Rothe T, et al Reproducibility of oscillometrically measured arterial stiffness indices: Results of the SAPALDIA 3 cohort study. Scand J Clin Lab Invest. 2015;75: 170–176. 10.3109/00365513.2014.993692 25594797

[9] Shirai K, Utino J, Otsuka K, Takata M. A Novel Blood Pressure-independent Arterial Wall Stiffness Parameter; Cardio-Ankle Vascular Index (CAVI). J Atheroscler Thromb. 2006;13: 101–107. 10.5551/jat.13.101 16733298

[10] Wassertheurer S, Mayer C, Breitenecker F. Modeling arterial and left ventricular coupling for non-invasive measurements. Simul Model Pract Theory. 2008;16: 988–997. 10.1016/j.simpat.2008.04.016

[11] Endes S, Bachler M, Li Y, Mayer C, Hanssen H, Hametner B, et al Feasibility of oscillometric aortic pressure and stiffness assessment using the VaSera VS-1500: comparison with a common tonometric method. Blood Press Monit. 2015;20: 273–279. 10.1097/MBP.0000000000000137 26065840

[12] Caviezel S, Dratva J, Schaffner E, Teynor A, Baumstark MW, Schindler C, et al Variability and reproducibility of carotid structural and functional parameters assessed with transcutaneous ultrasound—Results from the SAPALDIA Cohort Study. Atherosclerosis. 2013;231: 448–455. 10.1016/j.atherosclerosis.2013.10.010 24267265

[13] Teynor A, Caviezel S, Dratva J, Künzli N, Schmidt-Trucksäss A. An Automated, Interactive Analysis System for Ultrasound Sequences of the Common Carotid Artery. Ultrasound Med Biol. 2012;38: 1440–1450. 10.1016/j.ultrasmedbio.2012.03.015 22749339

[14] Caviezel S, Dratva J, Schaffner E, Schindler C, Endes S, Autenrieth CS, et al Carotid Stiffness and Physical Activity in Elderly—A Short Report of the SAPALDIA 3 Cohort Study. PLoS ONE. 2015;10: e0128991 10.1371/journal.pone.0128991 26035590 PMC4452761

[15] Van Bortel LM, Duprez D, Starmans-Kool MJ, Safar ME, Giannattasio C, Cockcroft J, et al Clinical applications of arterial stiffness, Task Force III: recommendations for user procedures. Am J Hypertens. 2002;15: 445–452. 10.1016/S0895-7061(01)02326-3 12022247

[16] Horinaka S, Yabe A, Yagi H, Ishimura K, Hara H, Iemua T, et al Comparison of Atherosclerotic Indicators Between Cardio Ankle Vascular Index and Brachial Ankle Pulse Wave Velocity. Angiology. 2008;60: 468–476. 10.1177/0003319708325443 19015165

[17] Vlachopoulos C, Aznaouridis K, Terentes-Printzios D, Ioakeimidis N, Stefanadis C. Prediction of Cardiovascular Events and All-Cause Mortality With Brachial-Ankle Elasticity Index: A Systematic Review and Meta-Analysis. Hypertension. 2012;60: 556–562. 10.1161/HYPERTENSIONAHA.112.194779 22733468

[18] Rosfors S, Hallerstam S, Jensen-Urstad K, Zetterling M, Carlström C. Relationship Between Intima-Media Thickness in the Common Carotid Artery and Atherosclerosis in the Carotid Bifurcation. Stroke. 1998;29: 1378–1382. 10.1161/01.STR.29.7.1378 9660390

[19] Riccioni G, D’Orazio N, Palumbo N, Bucciarelli V, di Ilio E, Bazzano LA, et al Relationship between plasma antioxidant concentrations and carotid intima-media thickness: the Asymptomatic Carotid Atherosclerotic Disease In Manfredonia Study. Eur J Cardiovasc Prev Rehabil. 2009;16: 351–357. 10.1097/HJR.0b013e328325d807 19384236

[20] Lorenz MW, Polak JF, Kavousi M, Mathiesen EB, Völzke H, Tuomainen T-P, et al Carotid intima-media thickness progression to predict cardiovascular events in the general population (the PROG-IMT collaborative project): a meta-analysis of individual participant data. The Lancet. 2012;379: 2053–2062. 10.1016/S0140-6736(12)60441-3 PMC3918517 22541275

[21] Vlachopoulos C, Xaplanteris P, Aboyans V, Brodmann M, Cífková R, Cosentino F, et al The role of vascular biomarkers for primary and secondary prevention. A position paper from the European Society of Cardiology Working Group on peripheral circulation: Endorsed by the Association for Research into Arterial Structure and Physiology (ARTERY) Society. Atherosclerosis. 2015;241: 507–532. 10.1016/j.atherosclerosis.2015.05.007 26117398

[22] Den Ruijter HM, Peters SE, Anderson TJ, et al Common carotid intima-media thickness measurements in cardiovascular risk prediction: A meta-analysis. JAMA. 2012;308: 796–803. 10.1001/jama.2012.9630 22910757

[23] den Ruijter HM, Peters S a. E, Groenewegen KA, Anderson TJ, Britton AR, Dekker JM, et al Common carotid intima-media thickness does not add to Framingham risk score in individuals with diabetes mellitus: the USE-IMT initiative. Diabetologia. 2013;56: 1494–1502. 10.1007/s00125-013-2898-9 23568273 PMC4523149

[24] Cobble M, Bale B. Carotid intima-media thickness: knowledge and application to everyday practice. Postgrad Med. 2010;122: 10–18. 10.3810/pgm.2010.01.2091 20107284

[25] Shirai K, Utino J, Otsuka K, Takata M. A Novel Blood Pressure-independent Arterial Wall Stiffness Parameter; Cardio-Ankle Vascular Index (CAVI). J Atheroscler Thromb. 2006;13: 101–107. 10.5551/jat.13.101 16733298

[26] Nakamura K, Tomaru T, Yamamura S, Miyashita Y, Shirai K, Noike H. Cardio-ankle vascular index is a candidate predictor of coronary atherosclerosis. Circ J. 2008;72: 598–604. 18362432 10.1253/circj.72.598

[27] Park J-B, Park HE, Choi S-Y, Kim MK, Oh B-H. Relation between Cardio-Ankle Vascular Index and Coronary Artery Calcification or Stenosis in Asymptomatic Subjects. J Atheroscler Thromb. 2013;20: 557–567. 10.5551/jat.15149 23524474

[28] Kim J-H, Rhee M-Y, Kim Y-S, Bae J-H, Nah D-Y, Kim Y-K, et al Brachial-ankle pulse wave velocity for the prediction of the presence and severity of coronary artery disease. Clin Exp Hypertens. 2013;36: 404–409. 10.3109/10641963.2013.846354 24164335

[29] Endes S, Schaffner E, Caviezel S, Dratva J, Autenrieth CS, Wanner M, et al Physical activity is associated with lower arterial stiffness in older adults: results of the SAPALDIA 3 Cohort Study. Eur J Epidemiol. 2015; 1–11. 10.1007/s10654-015-0076-8 26220521

[30] Endes S, Schaffner E, Caviezel S, Dratva J, Autenrieth CS, Wanner M, et al Long-term physical activity is associated with reduced arterial stiffness in older adults: longitudinal results of the SAPALDIA cohort study. Age Ageing. 2016;45: 110–115. 10.1093/ageing/afv172 26764400

[31] Yamashina A, Tomiyama H, Takeda K, Tsuda H, Arai T, Hirose K, et al Validity, reproducibility, and clinical significance of noninvasive brachial-ankle pulse wave velocity measurement. Hypertens Res. 2002;25: 359–364. 10.1291/hypres.25.359A 12135313

[32] Takaki A, Ogawa H, Wakeyama T, Iwami T, Kimura M, Hadano Y, et al Cardio-ankle vascular index is a new noninvasive parameter of arterial stiffness. Circ J. 2007;71: 1710–1714. 10.1253/circj.71.1710 17965489

[33] Izuhara M, Shioji K, Kadota S, Baba O, Takeuchi Y, Uegaito T, et al Relationship of Cardio-Ankle Vascular Index (CAVI) to Carotid and Coronary Arteriosclerosis. Circ J. 2008;72: 1762–1767. 10.1253/circj.CJ-08-0152 18802315

[34] Takaki A, Ogawa H, Wakeyama T, Iwami T, Kimura M, Hadano Y, et al Cardio-Ankle Vascular Index Is Superior to Brachial-Ankle Pulse Wave Velocity as an Index of Arterial Stiffness. Hypertens Res. 2008;31: 1347–1355. 10.1291/hypres.31.1347 18957805

[35] Okura T, Watanabe S, Kurata M, Manabe S, Koresawa M, Irita J, et al Relationship between Cardio-Ankle Vascular Index (CAVI) and Carotid Atherosclerosis in Patients with Essential Hypertension. Hypertens Res. 2007;30: 335–340. 10.1291/hypres.30.335 17541212

[36] Weber T, Wassertheurer S, Hametner B, Parragh S, Eber B. Noninvasive methods to assess pulse wave velocity: comparison with the invasive gold standard and relationship with organ damage. J Hypertens. 2015;33: 1023–1031. 10.1097/HJH.0000000000000518 25668350

[37] Torjesen A, Wang N, Larson MG, Hamburg NM, Vita JA, Levy D, et al Forward and backward wave morphology and central pressure augmentation in men and women in the Framingham Heart Study. Hypertension. 2014;64: 259–265. 10.1161/HYPERTENSIONAHA.114.03371 24866142 PMC4184952

