# Supplementary figures and images for: Associations of Novel and Traditional Vascular Biomarkers of Arterial Stiffness: Results of the SAPALDIA 3 Cohort Study
Source: PLoS One. 2016 Sep 29;11(9):e0163844. doi: 10.1371/journal.pone.0163844 (PMC5042378; doi:10.1371/journal.pone.0163844)

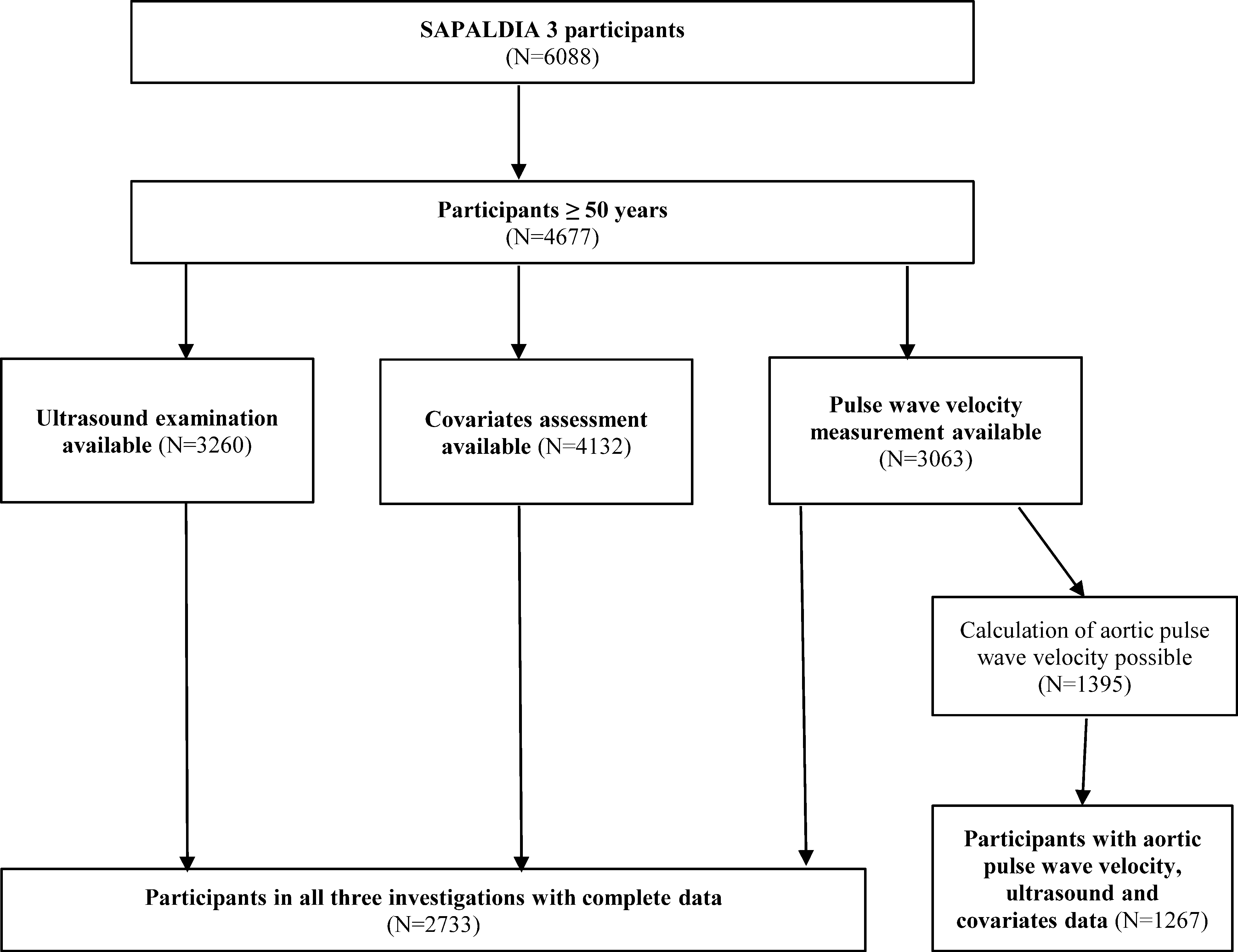

Supplement: S1 Fig — (TIF) [file pone.0163844.s001.tif]

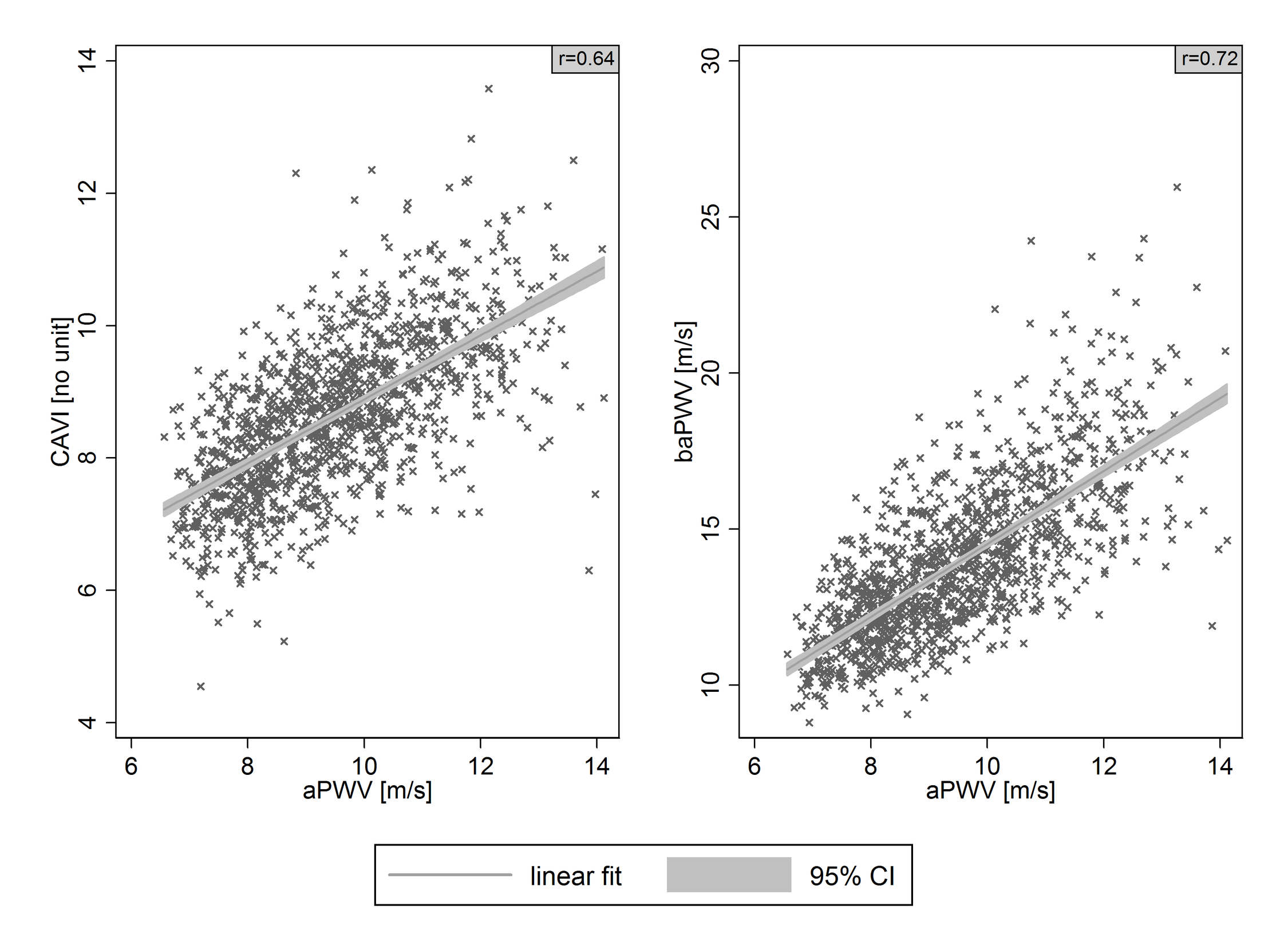

Supplement: S2 Fig — Scatter plots with linear prediction line (linear fit) and 95% confidence interval (95% CI). r denotes Pearson’s correlation coefficient. (TIF) [file pone.0163844.s002.tif]
