# Supplementary material for: Associations of Novel and Traditional Vascular Biomarkers of Arterial Stiffness: Results of the SAPALDIA 3 Cohort Study
Source: PLoS One. 2016 Sep 29;11(9):e0163844. doi: 10.1371/journal.pone.0163844 (PMC5042378; doi:10.1371/journal.pone.0163844)
Supplement: S1 File — (DOC) [file pone.0163844.s003.doc]

**Current SAPALDIA Team**

**Study directorate**: NM Probst-Hensch (PI; e/g); T Rochat (p), C Schindler (s), N Künzli (e/exp), JM Gaspoz (c)

**Scientific team**: JC Barthélémy (c), W Berger (g), R Bettschart (p), A Bircher (a), C Brombach (n), PO Bridevaux (p), L Burdet (p), Felber Dietrich D (e), M Frey (p), U Frey (pd), MW Gerbase (p), D Gold (e), E de Groot (c), W Karrer (p), F Kronenberg (g), B Martin (pa), A Mehta (e), D Miedinger (o), M Pons (p), F Roche (c), T Rothe (p), P Schmid-Grendelmeyer (a), D Stolz (p), A Schmidt-Trucksäss (pa), J Schwartz (e), A Turk (p), A von Eckardstein (cc), E Zemp Stutz (e).

**Scientific team at coordinating centers**: M Adam (e), I Aguilera (exp), S Brunner (s), D Carballo (c), S Caviezel (pa), I Curjuric (e), A Di Pascale (s), J Dratva (e), R Ducret (s), E Dupuis Lozeron (s), M Eeftens (exp), I Eze (e), E Fischer (g), M Foraster (e), M Germond (s), L Grize (s), S Hansen (e), A Hensel (s), M Imboden (g), A Ineichen (exp), A Jeong (g), D Keidel (s), A Kumar (g), N Maire (s), A Mehta (e), R Meier (exp), E Schaffner (s), T Schikowski (e), M Tsai (exp)

(a) allergology, (c) cardiology, (cc) clinical chemistry, (e) epidemiology, (exp) exposure, (g) genetic and molecular biology, (m) meteorology, (n) nutrition, (o) occupational health, (p) pneumology, (pa) physical activity, (pd) pediatrics, (s) statistics

**Research support**:
The Swiss National Science Foundation (grants no 33CS30-148470/1, 33CSCO-134276/1, 33CSCO-108796, , 324730_135673, 3247BO-104283, 3247BO-104288, 3247BO-104284, 3247-065896, 3100-059302, 3200-052720, 3200-042532, 4026-028099, PMPDP3_129021/1, PMPDP3_141671/1), the Federal Office for the Environment, the Federal Office of Public Health, the Federal Office of Roads and Transport, the canton's government of Aargau, Basel-Stadt, Basel-Land, Geneva, Luzern, Ticino, Valais, and Zürich, the Swiss Lung League, the canton's Lung League of Basel Stadt/ Basel Landschaft, Geneva, Ticino, Valais, Graubünden and Zurich, Stiftung ehemals Bündner Heilstätten, SUVA, Freiwillige Akademische Gesellschaft, UBS Wealth Foundation, Talecris Biotherapeutics GmbH, Abbott Diagnostics, European Commission 018996 (GABRIEL), Wellcome Trust WT 084703MA.

**Acknowledgements**The study could not have been done without the help of the study participants, technical and administrative support and the medical teams and field workers at the local study sites.
Local fieldworkers : Aarau: S Brun, G Giger, M Sperisen, M Stahel, Basel: C Bürli, C Dahler, N Oertli, I Harreh, F Karrer, G Novicic, N Wyttenbacher, Davos: A Saner, P Senn, R Winzeler, Geneva: F Bonfils, B Blicharz, C Landolt, J Rochat, Lugano: S Boccia, E Gehrig, MT Mandia, G Solari, B Viscardi, Montana: AP Bieri, C Darioly, M Maire, Payerne: F Ding, P Danieli A Vonnez, Wald: D Bodmer, E Hochstrasser, R Kunz, C Meier, J Rakic, U Schafroth, A Walder.

**Administrative staff**: N Bauer Ott, C Gabriel, R Gutknecht.
